# The effect of nanosilver-based preparation on microbiological quality of poultry litter

**DOI:** 10.5194/aab-66-421-2023

**Published:** 2023-12-11

**Authors:** Katarzyna Czyż, Zbigniew Dobrzański, Monika Kowalska-Góralska, Magdalena Senze, Anna Wyrostek

**Affiliations:** 1 Institute of Animal Breeding, Wrocław University of Environmental and Life Sciences, Wrocław, 51-630, Poland; 2 Department of Environment Hygiene and Animal Welfare, Wrocław University of Environmental and Life Sciences, Wrocław, 51-630, Poland

## Abstract

The study aimed to examine an effect of the preparation based on nanosilver suspension on mineral carrier on poultry litter microbiological profile. The study was conducted on Ross 308 broiler chickens. Three groups were formed, 84 birds in each. Preparation used in the study was composed of aqueous nanosilver suspension sprayed on mineral sorbent. Birds were maintained on straw-sawdust litter; the groups were differentiated due to preparation application (C – control without preparation, I – preparation applied once at the beginning, II – preparation added each week). Pooled litter samples were collected from the top layer of the litter (six samplings) in order to determine mesophilic bacteria count. Additionally, on the last day of the experiment litter samples were collected from three points (by drinker, feeder, pen corner) to analyze the total number of microorganisms, *Salmonella* spp., *Escherichia coli*, *Enterococci*, and molds. In the case of mesophilic bacteria count, the highest decrease was noted for group II. Total number of microorganisms determined in various points of the pen did not give clear relationship; in some cases even an increase was found. *Salmonella* spp. decreased as a result of preparation addition; the highest decrease was noted for samples collected by feeders. The results for *Escherichia coli* are not unequivocal. However, a decrease was found in the case of drinkers and feeders compared to control, especially in group II. An addition of preparation caused a decrease in *Enterococci*, especially for samples collected by feeders in group II. Similar tendency was found for molds. The study demonstrated that the preparation exhibits bactericidal properties. However, its effect varies depending on microorganism kind and sample collection point.

## 1 Introduction

Meat and meat products are considered as an important element of human diet, since they provide proteins, minerals, vitamins, and trace element contents. Chicken meat is one of the most popular kinds of meat in the world, which is the result of a few aspects. It is generally easily available and affordable, no religious issues affect its consumption, and it is considered healthy, easy to prepare, and responsive to consumer concerns. Production of poultry meat, especially broiler chickens, belongs to the most dynamic sectors of the meat industry, both in Poland and all over the world. Over the period of 2000–2016, the production of poultry meat increased by over 80 %, and the share of poultry meat on Polish market increased by nearly 30 %. Poland has become the leader in poultry meat production in the EU and one of the most significant exporters (Stańsko and Mikuła, 2017).

Thus, the poultry production sector is one of the most intensively developing meat production sectors in many countries. This is related to large density of birds in a small areas, which can be a significant source of microbiological contamination, of both litter and air in livestock buildings (Lonc and Plewa, 2010). This in turn constitutes an important problem both from a health-related and environmental point of view (Wójcik et al., 2010; Dumas et al., 2011; Kalus et al., 2017). The presence of pathogenic or relatively pathogenic microorganisms poses a risk of increased animal morbidity and the spread of microorganisms and parasite eggs in water and soil. This may lead to the inclusion of pathogenic organisms in the food chain, resulting in human infection (Bolan et al., 2010). Humans can be exposed to pathogens from poultry litter directly by contact with such a litter or indirectly due to polluter products derived from poultry (Chen and Jiang, 2014). Additionally, chicken litter, being a mixture of feces, feed, and material used as bedding and feathers, is one of the most popular and cheap organic fertilizers, characterized by significant nitrogen levels (Wilkinson et al., 2011; Kim et al., 2012; Chen and Jiang, 2014). Unfortunately, it is also a source of pathogens that can be potentially harmful to humans. Composting does not fully resolve this problem, as some pathogenic cells are considered to be able to persist the compositing process, and there is even the possibility of their regrowth (Lemunier et al., 2005; Kim et al., 2009). The danger concerns also water as it can be contaminated by runoff from poultry facilities as well as by lands fertilized with poultry manure (Thurston-Enriquez et al., 2005; Berry et al., 2007).

Therefore, it is important to seek effective disinfection methods in order to reduce, inter alia, the number of harmful microorganisms in animal production. One such method is the use of nanosilver suspension applied to mineral carriers (Nowakowski et al., 2011; Czyż et al., 2012, 2013). Biocidal properties of silver were recognized by humans already in ancient times, and it was used, for example, to preserve water (silver vessels or coins). With time, this element was also used as a medicinal product with antibacterial and antiseptic properties (Maillard and Hartemann, 2013). With the development of nanotechnology, various silver nanoparticle production methods have been elaborated, as it was found that substances in nanoform exhibit quite different and often more beneficial and useful properties, compared to corresponding bulk materials (Wijnhoven et al., 2009; Kowalska-Góralska et al., 2010). Nowadays, nanosilver has a wide range of applications (e.g., in medicine, pharmacy, cosmetology, chemical industry, agriculture, animal production), and many of these applications are focused just on its bactericidal properties.

Nanosilver affects the growth, movement, and reproduction of bacterial cells and blocks the respiration and metabolic reactions occurring within the cell. Nanosilver surrounds the bacterial cell with a tight layer, which makes it difficult for the cell to move, and the flagella are not able to exchange the genetic material. A bacterial cell surrounded by a layer of nanosilver also loses its ability to reproduce by cell division, because silver nanoparticles block the process of building new bacterial cell walls. The pattern of bacterial cell inactivation by colloidal silver is that it catalyzes the conversion of oxygen ions and molecular oxygen to atomic oxygen, which has the ability to sterilize. Preventing the formation of new cell walls and cell death by degrading the existing cell wall is accomplished by the reaction of atomic oxygen with the thiol groups of cysteine, which leads to the formation of sulfide bonds between amino acids. The nanosilver coating also damages the cell membrane by affecting its potential in such a way that it disrupts the sodium–potassium pumps, which in turn leads to a change in cell volume (swelling) and impairs the transport of sugars and amino acids into the cell. The catalytic properties of nanosilver lead to protein denaturation through the formation of free protons in bacterial cells, which cause the disulfide bonds to break. The disruption of the bacterial cell's respiration process involves disrupting the flow of electrons in the cell. Silver blocks metabolic reactions from occurring in cells. It inactivates the catalytic activity of enzymes by reacting with the - SH groups of enzymes. The mechanism of action of nanosilver on fungi and viruses is analogous to that described above on the example of bacterial cells. Nanosilver disrupts the water balance of fungi and affects the catalytic degradation of the lipid and protein substrates of viruses (Cho et al., 2005; Pal et al., 2007; Shrivastava et al., 2007; Rai et al., 2009; Li et al., 2010; Pulit-Prociak and Banach, 2016; Deshmukh et al., 2019).

The main task of nanosilver-based preparations in agriculture and animal production is the sterilization of instruments, equipment, and livestock facilities, as well as packaging and storage of both food and animal waste. In animal production nanosilver is used to disinfect animals, including the hooves and udders. Thanks to such a strong bactericidal, fungicidal, and deodorizing action of nanoparticles, they are used for disinfecting and protecting the surface of the ground, walls, and partitions of stable buildings and breeding facilities. It is used to disinfect and protect greenhouses, as well as containers for storing fodder and litter. It is important that preparations containing nanosilver are considered safe for humans and animals and that their efficacy in bacteria and fungi eliminating (as reported in the literature) is more than 99 % (Banach et al., 2007). Based on animal models, Rezvani et al. (2019) established that the nanosilver dose of <12 µ g mL ${}^{-1}$ with a specific surface area of 30 m ${}^{2}$ g ${}^{-1}$ , or < 25 mg kg ${}^{-1}$ body weight with a specific surface area of 20 m ${}^{2}$ g ${}^{-1}$ , is a safe threshold. On the other hand, according to Deshmukh et al. (2019), a dose of 1.56–6.25 g mL ${}^{-1}$ is safe for humans.

Thus, the aim of our study was to examine the effect of the preparation based on nanosilver suspension on mineral carrier on the microbiological profile of poultry litter.

## 2 Materials and methods

### 2.1 Animals and sampling

The animal material used in the study was Ross 308 line broiler chickens at the age of 2 weeks. The birds were randomly assigned to three research groups (84 birds in each, 15 heads m ${}^{-2}$ ), they were kept on the premises of the Faculty of Biology, Wrocław University of Environmental and Life Sciences, Poland, on straw-sawdust litter (1 : 1), and the individual groups differed due to the application of nanosilver-based preparation. The preparation used in the study was obtained spraying aqueous nanosilver suspension (Amepox, Łódź, Poland) on mineral sorbent, which was expanded vermiculite (Rominco Poland). An addition of 5 % of humodetrynite was applied in order to increase sorptive capacity of the preparation. Aqueous nanosilver suspension at a concentration of 1000 ppm was sprayed on a sorbent in an amount of 100 mL L ${}^{-1}$ of sorbent ( v/v ) at room temperature. This allowed us to obtain the friable preparation of a solid consistence, easy to use as a litter additive. Detailed characteristics of the preparation are presented in another study (Czyż et al., 2012). Briefly, the content of Ag in the preparation was about 253 ppm, while its content of the surface of the preparation particles based on X-ray analysis was 0.13 %. Scanning electron microscopy demonstrated that the preparation was characterized by an expanded structure in the form of parallel patches with numerous irregularities, resulting from the structure of vermiculite, which was not altered by aqueous nanosilver suspension addition (Czyż et al., 2012).

The following groups were formed: control (C) – no preparation addition; group I – preparation addition in amount of 15 L (i.e., about 3.7 kg) under litter surface – once at the beginning of the experiment; and group II – preparation addition in amount of 15 L mixed with litter, and next added again once a week during straw and sawdust addition. Straw and sawdust were also added on the same days in the control group and group I. The thickness of litter layer was maintained at a level of about 5–8 cm. The experiment lasted 4 weeks. The study was conducted in agreement with the 2nd Local Ethical Commission for the experiments on animals in Wrocław (agreement no. 115/2007 of 22 October 2007).

The birds were fed according to the standards of poultry feeding (Smulikowska and Rutkowski, 2005) with complete commercial mixtures of grower and then finisher type (Provimi Poland), and their body weight was determined at the beginning and at the end of the experiment, which allowed us to calculate the body weight gains during the experimental period. Also feed consumption rates were recorded.

Analyses of mesophilic bacteria count in pooled samples of upper litter layer were conducted on days 0, 5, 10, 15, 20, and 25 of the experiment according to the Polish standard PN-R-64791 at the Veterinary Laboratory VETLAB (Wrocław, Poland).

On the last day of the study, litter samples were additionally collected from three points (by drinker, feeder, and in pen corner) for quantitative and qualitative analyses of bacteria and fungi contents. These analyses were performed with the use of Merck reagents and substrates according to the methodology outlined in the following sections.

### 2.2 Determination of *Salmonella* spp. count

In order to determine *Salmonella* spp. count in the tested samples, the MPN (most probable number) method in the three-tube system was used; 1 % buffered peptone water was used for preliminary multiplication. Each time, a series of dilutions with the final dilution of 10–10 were used. Completed dilutions were incubated at 37 ${}^{\circ }$ C for 24 h. Selective multiplication was carried out with the use of a liquid selective medium according to Rappaport with an addition of tetrathionate and malachite green. The number of tubes in the series corresponded to the number of tubes with peptone water. The inoculated Rappaport medium was incubated at 43 ${}^{\circ }$ C for 24 h. BPL agar with brilliant green, phenolic red, and lactose and XLD agar with xylose, lysine, and deoxycholate as a solid medium were used in the calculations. Solid media were incubated at 37 ${}^{\circ }$ C for 24 h after inoculation The identification of *Salmonella* spp. was performed based on agglutination test with polyvalent HM serum. *Salmonella* spp. count was determined based on characteristic number and referring it to McCrady's tables (McCrady, 1918) for three technical replications and reading the MPN from them.

### 2.3 Determination of *Escherichia coli* count

In order to determine *Escherichia coli* count in the tested samples, the MPN method in the three-tube system was used. MacConkey broth was used in the first stage of isolation. Each time, a series of dilutions with the final dilution of 10–10 were used. Finished dilutions were incubated at 43 ${}^{\circ }$ C for 24 h. After the incubation period, inoculation on a solid medium – Tergitol-7-Agar with addition of TTC and ENDO agar – was made. The inoculated medium was incubated at 43 ${}^{\circ }$ C for 24 h. *E. coli* count was determined based on characteristic number and referring it to McCrady's tables for three replications and reading the MPN from them. Final identification was based on a set of API 20E biochemical microtests.

### 2.4 Determination of *Enterococci* count

In order to determine the number of *Enterococcus faecalis*, the MPN method in the three-tube system was used in the tested samples. In the first stage of isolation, broth with azide and glucose was used. Each time a series of dilutions with the final dilution of 10–10 were used. Finished dilutions were incubated at 37 ${}^{\circ }$ C for 24 h. After the incubation period, the samples were cultured on a solid medium – agar with kanamycin, eskulin, and azide. The inoculated medium was incubated at 37 ${}^{\circ }$ C for 24 h. The number of *E. faecalis* was specified by determining the characteristic number and referring it to McCrady's tables for three replications and reading the MPN from them. Final identification of *Enterococcus faecalis* was performed using the Phadebact D-Strep Test.

### 2.5 Calculation of total microbial count using the plate method

In order to calculate the number of microorganisms in the tested samples, a row of decimal dilutions in Ringer's solution was prepared. Next, subsequent dilutions were passaged into solid media suitable for the tested group of microorganisms:

- Nutritional agar was used to determine the total number of bacteria in culture.
- Potato dextrose agar (PDA) was used to determine the total number of fungi.

From each dilution, three parallel surface cultures were prepared on a suitable solid medium. The cultures were incubated at 37 ${}^{\circ }$ C for 24 h to determine the total number of bacteria, and the cultures for the total number of fungi were incubated at 25 ${}^{\circ }$ C for 72 h.

Two plates were selected for counting microorganisms, corresponding to each of two consecutive dilutions with a colony count not exceeding 300. After counting, the total bacterial count was determined according to the following formula (Banach et al., 2016):

$$L=\frac{\sum c}{(n_{1}+0.1n_{2})\times d},$$

where ∑c is the number of bacterial colonies on all plates, $n_{1}$ is the number of plates on which colonies from the first dilution were counted, $n_{2}$ is the number of plates on which colonies from the second dilution were counted, and d is the dilution index, lower than that at which counting was started.

In turn, the following formula was used to determine the total number of fungi:

$$L=\frac{\sum c}{\left(n\times d\right)},$$

where ∑c is the number of fungal colonies on all plates, n is the number of plates on which colonies were counted, and d is the dilution index, lower than that at which counting was started.

### 2.6 Statistical analysis

The results concerning rearing indicators and total number of microorganisms in upper litter layer were subjected to statistical analysis with an application of Statistica 13.0 software (StatSoft, Inc., Tulsa, OK, USA). Mean values and standard deviations were calculated. Normality of distribution was verified using the Shapiro–Wilk test when the sample size was <30 or Kolmogorov–Smirnov test when the sample size was ≥30 . Significance of differences between the groups was determined using Duncan's test (in the case of normal distribution) and Kruskal–Wallis test (lack of normal distribution) at a significance level of p<0.05 .

In turn, the results of quantitative and qualitative determination of the number of bacteria and fungi were subjected to statistical analysis using the SAS 9.2 PL program. Due to the discontinuous nature of the variable and lack of normal distribution of its values, the Kruskal–Wallis test and Dunn's non-parametric post hoc test were used. The analysis was performed assuming a significance level of p<0.05 and p<0.01 . The statistical analysis of the results did not show any significant differences when the experimental factors included the way of adding the nanosilver and the point of sampling. Only by including the number of replication in the group of experimental factors was it possible to determine the significance of differences between the individual results. The results were converted to a logarithmic measure ( log⁡10 ) in order to present them on a graph.

## 3 Results

The rearing indicators obtained in the study are presented in Table 1.

**Table 1:** Broiler chicken rearing indicators (average values).

Indicator	Control group (C)	Group I	Group II
Initial body weight (g)	400.3 ${}^{a}$ ± 10.9	399.3 ${}^{a}$ ± 11.9	420.4 ${}^{b}$ ± 11.5
Final body weight (g)	1730.0 ${}^{a}$ ± 114.1	1830.1 ${}^{b}$ ± 175.8	1842.1 ${}^{b}$ ± 185.6
Body weight gain (g)	1329.7 ${}^{a}$ ± 116.0	1430.8 ${}^{b}$ ± 176.8	1421.7 ${}^{b}$ ± 185.5
Feed consumption (grams per head per day)	118.7 ± 11.7	121.1 ± 13.8	120.1 ± 10.1
Feed utilization	2.16 ± 0.28	2.06 ± 0.35	2.06 ± 0.32
(kg/kg of body weight gain)			

${}^{a,b,c}$ – values in the lines marked with different letters differ at the significance level p<0.05 .

Initial body weight of chickens was at an average level of about 406 g, and this value at the end of the experiment reached 18 g. This was reflected in average body weight gains at a level of 1394 g. Feed consumption was 120 g per head per day on average, while the value expressed in kg/kg of body weight was 2.07. No statistically significant differences between the groups were noted in the case of feed consumption and utilization.

The content of mesophilic bacteria in pooled samples of the upper litter layer depending on the group is shown in Table 2.

The highest reduction in mesophilic bacteria count compared to the control group was observed in group II, where the nanosilver-based preparation was mixed with litter and added at each bedding. The reduction was in the range from about 33 % on the fifth day to 88 % on the last day of the experiment. Slightly lower reduction was observed in group I, where the preparation was applied only once under litter at the beginning of the experiment, and it ranged from 32 % to 53 %. Except day 0, i.e., the beginning of the experiment, statistically significant differences were noted between the groups.

**Table 2:** Total mesophilic bacteria count in the upper litter layer (cfu × 10 ${}^{8}$ ) – pooled samples (mean ± SD).

Day	Control group (C)	Group I	Group II
0	16.53 ± 1.68	14.79 ± 2.00	13.25 ± 1.95
5	17.76 ${}^{a}$ ± 1.74	12.04 ${}^{b}$ ± 1.99	11.91 ${}^{b}$ ± 1.82
10	19.62 ${}^{a}$ ± 1.93	10.24 ${}^{b}$ ± 1.97	9.03 ${}^{b}$ ± 2.00
15	21.02 ${}^{a}$ ± 2.54	9.78 ${}^{b}$ ± 2.00	7.12 ${}^{b}$ ± 1.93
20	20.35 ${}^{a}$ ± 2.26	10.12 ${}^{b}$ ± 1.95	4.06 ${}^{c}$ ± 1.21
25	19.12 ${}^{a}$ ± 2.06	8.91 ${}^{b}$ ± 1.68	2.34 ${}^{c}$ ± 0.57

${}^{a,b,c}$ – values in the lines marked with different letters differ at the significance level p<0.05 .

**Figure 1:**
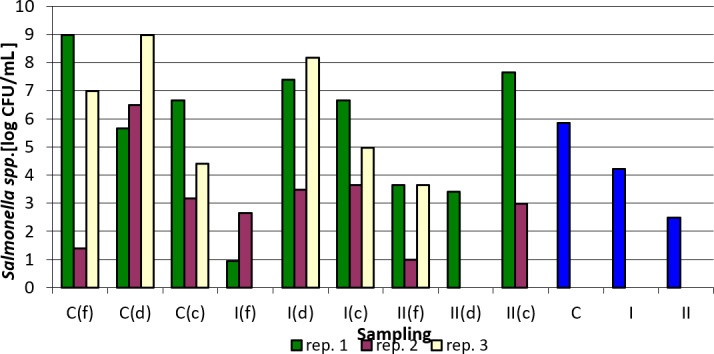
*Salmonella* spp. counts depending on sampling point and replication. Explanations: C – control group, I – group I, II – groups II, f – feeder, d – drinker, c – corner, and blue bars – mean values for groups C, I, and II.

**Figure 2:**
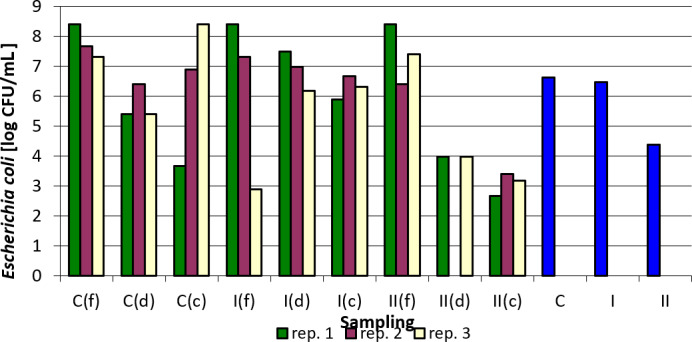
*Escherichia coli* counts depending on sampling point and replication. Explanations: C – control group, I – group I, II – groups II, f – feeder, d – drinker, c – corner, and blue bars – mean values for groups C, I, and II.

**Figure 3:**
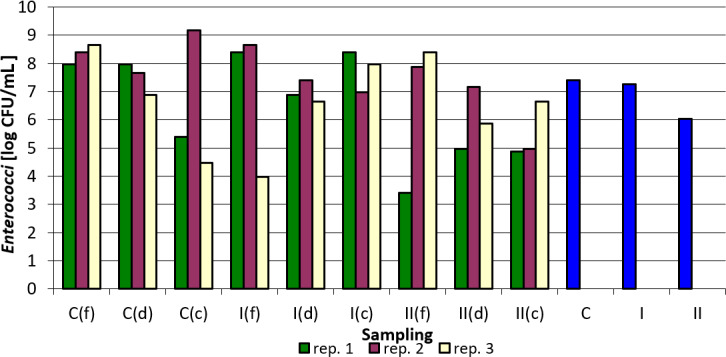
*Enterococcus faecalis* count depending on sampling point and replication. Explanations: C – control group, I – group I, II – groups II, f – feeder, d – drinker, c – corner, and blue bars – mean values for groups C, I, and II.

**Figure 4:**
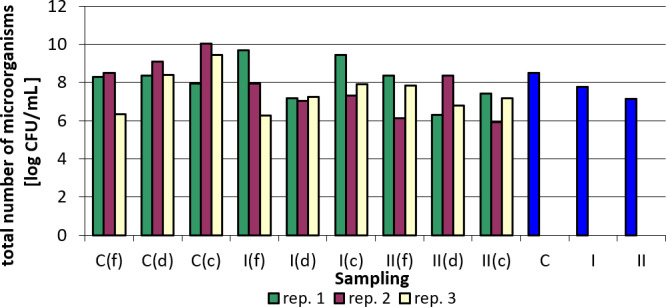
Total bacterial count depending on sampling point and replication. Explanations: C – control group, I – group I, II – groups II, f – feeder, d – drinker, c – corner, and blue bars – mean values for groups C, I, and II.

**Figure 5:**
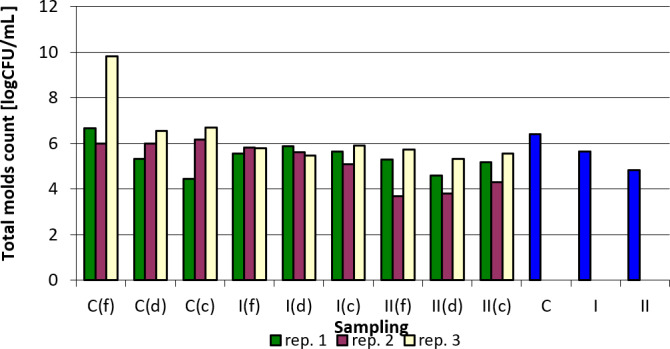
Total count of mold fungi depending on sampling point and replication. Explanations: C – control group, I – group I, II – groups II, f – feeder, d – drinker, c – corner, and blue bars – mean values for groups C, I, and II.

Tables 3–7 and Figs. 1–5 summarize the results of quantitative and qualitative analysis of bacteria and fungi in litter samples collected on the last day of the study at three points of the experimental pens (i.e., by the drinker, by the feeder, and in the corners of the pens).

The number of *Salmonella* spp. in the tested samples was the lowest in group I, feeders, replication 1, and the highest in the control group, feeders, replication 1 and control group, drinkers, replication 3. In most cases, the addition of the preparation to the litter resulted in a decrease in *Salmonella* counts. It was the most pronounced in case of samples collected at the feeders, where it reached even the order of 10 ${}^{8}$ in the case of replication 1 between the control group and group I or the order of 10 ${}^{3}$ in the case of replication 3 between the control group and group II. The smallest differences were observed in all replications for samples collected in the corners, where the changes recorded were small, and in some cases even an increase in the count was noted (e.g., $4.5\times 10^{6}$ in groups C and I and $4.5\times 10^{7}$ in group II in replication 1). The samples collected by drinkers were generally characterized by a decrease in *Salmonella* counts in the experimental groups compared to the control, except for group I in replication 1, where there was an increase compared to the control group (Table 3).

**Table 3:** *Salmonella* spp. count by sample type and replication (cfu mL ${}^{-1}$ ).

*Salmonella* spp. – replication 1			
Point	Control group (C)	Group I	Group II
Feeders	9.5 × 10 ${}^{8\;A}$	0.9 × 10 ${}^{1\;B}$	4.5 × 10 ${}^{3\;C}$
Drinkers	4.5 × 10 ${}^{5\;A}$	2.5 × 10 ${}^{7\;B}$	2.5 × 10 ${}^{3\;C}$
Corners	4.5 × 10 ${}^{6\;A}$	4.5 × 10 ${}^{6\;A}$	4.5 × 10 ${}^{7\;B}$
*Salmonella* spp. – replication 2			
Point	Control group (C)	Group I	Group II
Feeders	2.5 × 10 ${}^{1\;A,a}$	4.5 × 10 ${}^{2\;B}$	2.0 × 10 ${}^{1\;A,C,b}$
Drinkers	30.0 × 10 ${}^{5\;A}$	30.0 × 10 ${}^{2\;B}$	n.d.
Corners	15.0 × 10 ${}^{2\;A,a}$	4.5 × 10 ${}^{3\;B}$	9.5 × 10 ${}^{2\;A,C,b}$
*Salmonella* spp. – replication 3			
Point	Control group (C)	Group I	Group II
Feeders	9.5 × 10 ${}^{6\;A}$	n.d.	4.5 × 10 ${}^{3\;B}$
Drinkers	9.5 × 10 ${}^{8\;A}$	15.0 × 10 ${}^{7\;B}$	n.d.
Corners	2.5 × 10 ${}^{4\;a}$	9.5 × 10 ${}^{4\;b}$	n.d.

n.d. – not detected. ${}^{A,B,C}$ – values in the lines marked with different letters differ between the groups at the significance level p<0.01 . ${}^{a,b,c}$ – values in the lines marked with different letters differ between the groups at the significance level p<0.05 .

Taking into account the values averaged over sampling sites and replications, it can be definitely concluded that the applied preparation reduced the number of *Salmonella* in the groups where it was applied (Fig. 1).

The results for *Escherichia coli* counts indicate that the nanosilver-based preparation used did not cause a significant decrease in their values. A decrease was observed between group C and group II in replication 1 for samples collected by drinkers, between group C and group II in the same replication for samples collected in the corners, between group C and group II in replication 2 for samples taken by feeders, and between group C and group II for samples taken in the corners. The most pronounced differences were noted in replication 3 between the control group and group I for samples taken by feeders, between group C and group II for samples taken by drinkers, and between group C and group II for samples taken in the corners (Table 4).

**Table 4:** *Escherichia coli* count by sample type and replication (cfu mL ${}^{-1}$ ).

*Escherichia coli* – replication 1			
Point	Control group (C)	Group I	Group II
Feeders	2.5 × 10 ${}^{8}$	2.5 × 10 ${}^{8}$	2.5 × 10 ${}^{8}$
Drinkers	2.5 × 10 ${}^{5\;A}$	30.0 × 10 ${}^{6\;B}$	9.5 × 10 ${}^{3\;C}$
Corners	4.5 × 10 ${}^{3\;A}$	7.5 × 10 ${}^{5\;B}$	4.5 × 10 ${}^{2\;C}$
*Escherichia coli* – replication 2			
Point	Control group (C)	Group I	Group II
Feeders	4.5 × 10 ${}^{7\;A,a}$	2.0 × 10 ${}^{7\;A,b}$	2.5 × 10 ${}^{6\;B}$
Drinkers	2.5 × 10 ${}^{6\;a}$	9.5 × 10 ${}^{6\;b}$	n.d.
Corners	7.5 × 10 ${}^{6\;A,a}$	4.5 × 10 ${}^{6\;A,b}$	2.5 × 10 ${}^{3\;B}$
*Escherichia coli* – replication 3			
Point	Control group (C)	Group I	Group II
Feeders	20.0 × 10 ${}^{6\;A}$	7.5 × 10 ${}^{2\;B}$	2.5 × 10 ${}^{7\;C}$
Drinkers	2.5 × 10 ${}^{5\;A,a}$	15.0 × 10 ${}^{5\;A,b}$	9.5 × 10 ${}^{3\;B}$
Corners	2.5 × 10 ${}^{8\;A}$	20.0 × 10 ${}^{5\;B}$	15.0 × 10 ${}^{2\;C}$

n.d. – not detected. ${}^{A,B,C}$ – values in the lines marked with different letters differ between the groups at the significance level p<0.01 . ${}^{a,b,c}$ – values in the lines marked with different letters differ between the groups at the significance level p<0.05 .

Figure 2 shows the logarithmically transformed values for the number of *E. coli* in the samples tested, as well as the averaged values. In spite of considerable variability within individual sampling and replications, the effect of the nanosilver-based preparation on the reduction of *E. coli* counts in comparison to the control group is clearly visible, especially in group II where a higher total dose of the formulation was administered.

The count of *Enterococcus faecalis* in the tested samples was the lowest in group C, corners, replication 3, and the highest in the control group, corners, replication 3. The addition of the examined preparation to the litter resulted in a decrease in their counts in most cases. It was the most pronounced between the control group and group I in the case of samples collected by feeders in replication 1 and in replication 3 also for samples collected by feeders between group C and group I. For samples taken by drinkers, a decrease in *Enterococcus faecalis* count was observed between the control group and groups I and II in replication 1, in replication 2 between groups C and group II, and in replication 3 between the control group and group II. The smallest differences, or even increases, were observed in all replications for samples taken at the corners of the pens (Table 5).

**Table 5:** *Enterococcus faecalis* count by sample type and replication (cfu mL ${}^{-1}$ ).

*Enterococcus faecalis* – replication 1			
Point	Control group (C)	Group I	Group II
Feeders	9.5 × 10 ${}^{7\;A}$	2.5 × 10 ${}^{8\;B}$	2.5 × 10 ${}^{3\;C}$
Drinkers	9.5 × 10 ${}^{7\;A}$	7.5 × 10 ${}^{6\;B}$	9.5 × 10 ${}^{4\;C}$
Corners	2.5 × 10 ${}^{5\;A}$	2.5 × 10 ${}^{8\;B}$	7.5 × 10 ${}^{4\;C}$
*Enterococcus faecalis* – replication 2			
Point	Control group (C)	Group I	Group II
Feeders	2.5 × 10 ${}^{8\;A,a}$	4.5 × 10 ${}^{8\;A,b}$	7.5 × 10 ${}^{7\;B}$
Drinkers	4.5 × 10 ${}^{7\;A,a}$	2.5 × 10 ${}^{7\;A,b}$	15.0 × 10 ${}^{6\;B}$
Corners	15.0 × 10 ${}^{8\;A}$	9.5 × 10 ${}^{6\;B}$	9.5 × 10 ${}^{4\;C}$
*Enterococcus faecalis* – replication 3			
Point	Control group (C)	Group I	Group II
Feeders	4.5 × 10 ${}^{8\;A,a}$	9.5 × 10 ${}^{3\;B}$	2.5 × 10 ${}^{8\;A,b}$
Drinkers	7.5 × 10 ${}^{6\;A,a}$	4.5 × 10 ${}^{6\;A,a}$	7.5 × 10 ${}^{5\;B}$
Corners	30.0 × 10 ${}^{3\;A}$	9.5 × 10 ${}^{7\;B}$	4.5 × 10 ${}^{6\;C}$

${}^{A,B,C}$ – values in the lines marked with different letters differ between the groups at the significance level p<0.01 . ${}^{a,b,c}$ – values in the lines marked with different letters differ between the groups at the significance level p<0.05 .

As in the case of *Salmonella* spp. and *Escherichia coli*, the figure below clearly shows the effect of the applied nanosilver-based preparation on the reduction of *Enterococcus faecalis* count in the case of group II compared to the control group (Fig. 3).

Total bacterial counts were the lowest in corner samples in group II in replication 2, and the highest in corner samples in the control group in replication 2. When analyzing the results for individual replications and sampling points, it is difficult to find a clear relationship. There was a clear reduction in the total bacterial count in samples taken by drinkers. However, for the samples collected by feeders, even an increase in the total bacterial count was observed compared to the control group (group I and II in replication 1, group II in replication 3), while in the case of the corners the increase occurred only in the case of group I compared to the control group in replication 1 (Table 6).

**Table 6:** Total bacterial count by sample type and replication (cfu mL ${}^{-1}$ ).

Total bacterial count – replication 1			
Point	Control group (C)	Group I	Group II
Feeders	1.95 × 10 ${}^{8\;A}$	4.97 × 10 ${}^{9\;A}$	2.27 × 10 ${}^{8\;C}$
Drinkers	2.32 × 10 ${}^{8\;A}$	1.48 × 10 ${}^{7\;B}$	1.95 × 10 ${}^{6\;C}$
Corners	8.36 × 10 ${}^{7\;A}$	2.81 × 10 ${}^{9\;B}$	2.60 × 10 ${}^{7\;C}$
Total bacterial count – replication 2			
Point	Control group (C)	Group I	Group II
Feeders	3.09 × 10 ${}^{8\;A}$	8.72 × 10 ${}^{7\;B}$	1.36 × 10 ${}^{6\;C}$
Drinkers	1.19 × 10 ${}^{9\;A}$	1.12 × 10 ${}^{7\;B}$	2.27 × 10 ${}^{8\;C}$
Corners	1.10 × 10 ${}^{10\;A}$	2.10 × 10 ${}^{7\;B}$	8.45 × 10 ${}^{5\;C}$
Total bacterial count – replication 3			
Point	Control group (C)	Group I	Group II
Feeders	2.24 × 10 ${}^{6\;A}$	1.85 × 10 ${}^{6\;B}$	6.73 × 10 ${}^{7\;C}$
Drinkers	2.47 × 10 ${}^{8\;A}$	1.68 × 10 ${}^{7\;B}$	6.09 × 10 ${}^{6\;C}$
Corners	2.76 × 10 ${}^{9\;A}$	8.00 × 10 ${}^{7\;B}$	1.53 × 10 ${}^{7\;C}$

${}^{A,B,C}$ – values in the lines marked with different letters differ between the groups at the significance level p<0.01 .

Figure 4 presents logarithmically transformed values for the total number of bacteria in the samples analyzed and the values averaged for sampling point and replications. There is a clear downward trend in the total bacterial count in the groups where the nanosilver-based preparation was added (Fig. 4).

The results concerning the total count of mold fungi in the samples analyzed depending on the point of sampling and replication are summarized in Table 7. Apart from replication 1, where in the case of samples collected in the corners, a slight increase in the total number of molds was observed, a clearly positive effect of the applied preparation on the reduction of the total mold fungi count in the samples examined can be seen. For all analyzed samples the values were the lowest in group II, replication 2, samples collected by feeders, and the highest in the control group, replication 3, samples collected by feeders. The reduction in group II (i.e., in the group in which a higher dose of nanosilver was applied in total) was generally greater compared to group I, where the preparation was applied once at the beginning of the experiment (Table 7).

**Table 7:** Total count of mold fungi by sample type and replication (cfu mL ${}^{-1}$ ).

Total count of mold fungi – replication 1			
Point	Control group (C)	Group I	Group II
Feeders	4.50 × 10 ${}^{6\;A}$	3.65 × 10 ${}^{5\;B}$	2.00 × 10 ${}^{5\;C}$
Drinkers	2.10 × 10 ${}^{5\;A}$	7.35 × 10 ${}^{5\;B}$	3.90 × 10 ${}^{4\;C}$
Corners	2.75 × 10 ${}^{4\;A}$	4.50 × 10 ${}^{5\;B}$	1.50 × 10 ${}^{5\;C}$
Total count of mold fungi – replication 2			
Point	Control group (C)	Group I	Group II
Feeders	1.00 × 10 ${}^{6\;A}$	6.50 × 10 ${}^{5\;B}$	5.00 × 10 ${}^{3\;C}$
Drinkers	1.00 × 10 ${}^{6\;A}$	4.00 × 10 ${}^{5\;B}$	6.50 × 10 ${}^{3\;C}$
Corners	1.50 × 10 ${}^{6\;A}$	1.20 × 10 ${}^{5\;B}$	2.00 × 10 ${}^{4\;C}$
Total count of mold fungi – replication 3			
Point	Control group (C)	Group I	Group II
Feeders	6.65 × 10 ${}^{9\;A}$	6.30 × 10 ${}^{5\;B}$	5.35 × 10 ${}^{5\;C}$
Drinkers	3.50 × 10 ${}^{6\;A}$	2.85 × 10 ${}^{5\;B}$	2.05 × 10 ${}^{5\;C}$
Corners	5.00 × 10 ${}^{6\;A}$	8.05 × 10 ${}^{5\;B}$	3.50 × 10 ${}^{5\;C}$

${}^{A,B,C}$ – values in the lines marked with different letters differ between the groups at the significance level p<0.01 .

Figure 5 shows the total number of mold fungi (logarithmically transformed values) in relation to sampling point and replication. From the summary (C, I, II), the beneficial effect of the nanosilver-based preparation in terms of reducing the total number of molds in the litter can be concluded.

## 4 Discussion

Although some significant influence of the applied preparation based on vermiculite and aqueous suspension of nanosilver on the presented rearing indicators was observed (body weight and gains), it should be born in mind that the productivity of birds consists of a number of factors, and the too few observations do not allow conclusions to be drawn in this regard (Hafez and Hauck, 2005; Grashorn and Serini, 2006; Herbut, 2009).

All microbiological analyses (total bacteria count, *Salmonella* spp., *Escherichia coli*, *Enterococcus faecalis*, total mold fungi count) carried out in the present study, despite differences in results between individual sampling points (feeders, drinkers, corners) and repetitions, demonstrated a clear effect of the nanosilver-based preparation added to the litter on the reduction of microorganism level. This reduction was greater in group II, in which the preparation was added in the form mixed with litter at each bedding, but also a single application of the preparation under the litter at the beginning of the experiment resulted in lower values of the analyzed microbiological parameters.

In the available literature, many works can be found on the use of nanosilver to reduce the number of microorganisms in the aquatic environment, soil, or in the food industry, but they mainly concern experimental studies (e.g., Kopeikin, 2001; Alt et al., 2004; Damm et al., 2007; Abdi et al., 2008; Chau et al., 2008). However, no work has been found on the biocidal effect of formulations that are a combination of nanosilver and mineral sorbent in animal housing environments, as demonstrated in this paper. Therefore, it is difficult to compare our results with literature reports.

Prior to the experiment presented in this paper, we conducted an in vitro study using various nanosilver-based preparations, among others, the preparation applied in this study. Preparations based on nanosilver aqueous and alcohol solution were applied to various mineral sorbents (vermiculite, halloysite, and bentonite) and applied to poultry litter in laboratory conditions. The highest effectiveness was found for preparation based on vermiculite and aqueous nanosilver suspension, and the reduction in total bacteria count was on a level of about 84 % (Czyż et al., 2018). This was also the basis for the choice of preparation used in this experiment. Parallel tests were conducted by Dobrzański et al. (2010) on sheep manure using, inter alia, the preparation applied in this study, and it showed that its addition at a level of 10 % resulted in the reduction in total bacterial count in manure mixed with litter on a level of about 84 %.

The available literature contains quite a few reports on microbiological contamination of litter in poultry. Mituniewicz et al. (2008), for example, conducted a study to evaluate the effect of selected additives on reducing microbial contamination in poultry housing. The authors achieved a 58.2 % reduction in total bacterial count using products containing natural phosphates, copper in inorganic form, iron, and white clay. In another study, the same authors applied calcium peroxide and noted its effect on litter microflora stabilization (Mituniewicz et al., 2016). Also Lopes et al. (2013) conducted the study using quicklime (CaO) in poultry litter in order to reduce pathogenic bacteria count. However, most studies available in the literature present the results of litter treatment after production cycle, without the presence of the birds (e.g., Payne et al., 2005; Lopes et al., 2015).

On the other hand, regarding the antimicrobial activity of nanosilver, the literature is extensive; however, there is a lack of research on its application for disinfection of livestock housing. Shrivastava et al. (2007) demonstrated growth inhibition of various bacterial strains under laboratory conditions using silver nanoparticles. For *E. coli*, for example, the reduction ranged from 60 % depending on the concentration of nanosilver. Similarly, Yoon et al. (2007) observed a dose-dependent inhibition of bacterial growth using silver nanoparticles, but they concluded that the size of the nanoparticles could also affect the antibacterial activity. This is also reported by other authors who claim that smaller nanosilver particles have a larger surface area and thus demonstrate a better antibacterial effect compared to larger nanoparticles (Kumar et al., 2020).

The action of nanosilver leads to the degradation of cells of bacteria, fungi, and even viruses. This action is similar to that of antibiotics. The problem with the use of antibiotics is that new strains of bacteria are constantly emerging and becoming resistant to their effects. On the other hand, there is no information in the literature about the possible development of bacterial resistance to silver nanoparticles, which is probably due to multimode several-level action of these particles on microbial cells (Kamat and Kumari, 2023). It should be also emphasized that gram-positive bacteria like, e.g., *Enterococci* are more resistant to nanosilver particles compared to gram-negative ones, e.g., *E. coli* or *Salmonella* spp. (Zarei et al., 2014; Piskaeva et al., 2017), which was also demonstrated in our study, as well as in the study by Banach et al. (2016). The cell wall of gram-negative bacteria is composed of a thin inner peptidoglycan layer and an outer liposaccharide layer, while the cell wall of gram-positive bacteria contains a negatively charged peptidoglycan layer, which is much thicker than in gram-negative microorganisms, which makes the process of silver nanoparticles attachment and penetration through cell wall of gram-positive bacteria more difficult. Lower sensitivity of gram-positive bacteria to silver nanoparticles is also explained by higher thickness of cell wall and the presence of teichoic acids limiting silver nanoparticle absorption (Pazos-Ortiz et al., 2017; Dominguez et al., 2020; Feng et al., 2000; Popova and Ignatov, 2023).

Certainly, the potential hazard of nanoparticles, including silver ones, should be considered. The toxicity of silver nanoparticles may be affected by numerous factors, including their physicochemical features, exposure degree, or scope of their biological activity. Their widespread use may lead to an accumulation in all components of the environment. However, the degree of this accumulation and far-reaching effects are difficult to predict (Pulit-Prociak and Banach, 2016).

## 5 Conclusions

The study conducted demonstrated that preparations based on silver nanoparticles can be an alternative for an application in poultry, and presumably other livestock, litter. Application of nanosilver-based preparation results in the reduction of the share of possibly pathogenic bacteria, thus contributing to an increased safety of litter, as well as better health status of animals not exposed to high bacterial counts during rearing. This in turn should be of importance also for humans and consumers of poultry meat. However, further studies also concerning possible risks resulting from nanosilver application in poultry are needed.

## Data Availability

Data will be made available upon reasonable request.
